# Methodological Factors Involved in the Study of Temporal Binding Using the Open Source Software Labclock Web

**DOI:** 10.3389/fpsyg.2020.01040

**Published:** 2020-05-27

**Authors:** Carmelo P. Cubillas, Íñigo Landáburu, Helena Matute

**Affiliations:** ^1^Facultad de Psicología, Universidad Autónoma de Madrid, Madrid, Spain; ^2^Facultad de Ciencias de la Salud y de la Educación, Universidad a Distancia de Madrid, Madrid, Spain; ^3^Facultad de Psicología y Educación, Universidad de Deusto, Bilbao, Spain

**Keywords:** temporal binding, intentional binding, Libet’s clock, Labclock Web, online experiments

## Abstract

Temporal binding occurs when an action and an outcome that follows it after a short period of time are judged as occurring closer to each other in time than they actually are. This effect has often been studied using Libet’s clock methodology. Garaizar et al. (2016) presented Labclock Web, a free HTML5 open source software that allows researchers to conduct temporal binding and other experiments using Libet’s clock through the Internet. The purpose of the three experiments presented here was to test how certain methodological modifications in the Labclock Web task could impact the temporal binding effect. In comparison with the original study, we aimed to: (a) reduce the interval between action and outcome in the delayed condition to 100 ms, instead of 500, (b) present the two types of trials, immediate and delayed, in two separate consecutive blocks, instead of intermixed, (c) use a visual, rather than auditory, outcome following the action, and (d) reduce the number of trials. In addition to its potential theoretical implications, the results confirm that Labclock Web is a useful and reliable tool for conducting temporal binding experiments and that it is well suited to measure temporal binding effects in a broad range of situations.

## Introduction

As humans, we have the ability to modify the environment through our actions; this is why we are agents. The study of action-related phenomena such as the perception of our action, our will of action, the sense of agency produced when we act, or the awareness of the action-effect relationship, among others, has captured researchers’ attention during the latest decades. In one of the most well-known studies, Haggard et al. (2002a, b) explored the perceived time of voluntary actions and their effects. Based on a procedure developed by Libet et al. (1983) these authors found that when an intentional action produced the onset of a tone after 250 ms, experimental participants tended to perceive action and tone closer in time than they actually were. However, this binding effect did not occur when actions were unintentional (triggered by transcranial magnetic stimulation). For this reason, they referred to this effect as *intentional binding*. A few years later, Buehner and Humphreys (2009; see also Moore et al., 2009) found that this action-effect binding occurred only when participants perceived a causal relationship between the two events. Thus, they named it *causal binding* instead of intentional binding. Regardless of whether the key factor in binding is causality, intentionality, or some other factor such as expectancy (Engbert and Wohlschläger, 2007), this effect has received a considerable amount of interest in recent years and has been shown to be robust. Throughout this article, we will refer to this effect by using the more neutral term temporal binding.

Although several procedures have been used to study this effect (e.g., Mitchell et al., 2005; Stetson et al., 2006; Zhao et al., 2013), Libet’s clock has been probably the one most frequently used. Libet’s clock consists of the presentation of a spot that rotates around a clock-face at a speed of one revolution each 2,560 ms. Typically, participants are requested to perform an action such as, for instance, pressing the spacebar while the dot is moving. Immediately after the action (or some milliseconds after it) another stimulus, generally a tone, may appear. When the dot has finished rotating around the sphere, participants are asked to indicate on the clock-face the precise location of the dot when they performed the action or, in some other cases, when the tone occurred. As mentioned above, it is typically observed that participants tend to misestimate the occurrence of these events, action and tone. They tend to report that the two events occurred closer to each other in time than was actually the case. At the same time that the participants are conducting the task in Libet’s clock, their brain activity can also be monitored, for example, through electroencephalography. This allows experimenters to know, for example, the moment at which the participants start to perform the action at brain level, and compare this time to that in which the participants say they felt compelled to perform the action. This has important implications for the understanding of our awareness and free will (e.g., Libet et al., 1983; Libet, 1985; Haggard and Eimer, 1999). Moreover, the study of temporal binding with Libet’s clock has also been used as an indirect measurement of the sense of agency (Haggard et al., 2002a, b). This can be defined as the sense of control that people have over their actions and their consequences (Moore, 2016), and it is relevant in the study of psychological processes, in addition to certain mental disorders (e.g., Frith et al., 2000; Blakemore et al., 2002). To sum up, Libet’s clock is a powerful and popular procedure for conducting experiments in many interesting fields of psychology and neuroscience.

However, until very recently, there was no standardized version of Libet’s clock that allowed for an easy and homogeneous programming of the experiments. Garaizar et al. (2016) aimed to solve this problem. They presented Labclock Web, an open-source, HTML5 tool, to program and conduct experiments based on Libet’s procedure. According to these authors, Labclock Web offers three main advantages in comparison to other experimental software. First, it is a free open source tool, which allows researchers to modify freely the configuration needed to adapt the program to their own experimental design, and to detect potential errors and correct them. Second, it can be easily programmed by non-experts. Third, Labclock Web allows for conducting experiments online, which enables experimenters to have access to a wide number of participants, and therefore saves the costs associated with resources such as experimentation cabins or computers, which are not always available, and facilitates the recruitment of heterogeneous samples. Using this software, Garaizar et al. (2016) replicated the temporal binding effect. They showed that when the tone occurred 500 ms after the action, participants shifted their judgments of the time of occurrence of the action toward the occurrence of the tone; in contrast, their judgments of the time of action were relatively accurate when the tone was presented immediately after the action. In conclusion, they showed that Labclock Web was a reliable tool that can be used to conduct experiments based on Libet’s clock. However, in our view, there are some methodological issues in temporal binding experiments that remain unexplored both in the study of Garaizar et al. (2016) as well as in previous studies. The purpose of the experiments presented here is to explore the effect of certain methodological changes on temporal binding effects using Labclock Web. These methodological modifications are introduced with the aim of testing and expanding the utility of the Labclock Web open-source software.

We introduce also a change with respect to standard experiments in the literature (e.g., Haggard et al., 2002a; Moore and Haggard, 2007) that has to do with a change in the control conditions. In many experiments on temporal binding, two or three types of trials are presented in a within-participants manipulation: (a) operant trials, in which a tone is presented some milliseconds after the participant’s action, and (b) one or two baseline conditions in which only the action (with no tone), or only the tone (with no action) occur (but see also Wohlschläger et al., 2003; Stetson et al., 2006; Obhi and Hall, 2011; Desantis et al., 2012; Matute et al., 2017, for experiments in which control conditions were different). In this standard procedure the difference between the subjective time and the actual time for the action and for the tone in the operant condition are compared to their respective action-alone or tone-alone conditions. If the perception of the action is displaced toward the outcome in the operant trials as compared to the trials in which outcome is not presented, then it is concluded that action binding has occurred. Conversely, if the perception of the outcome is displaced toward the action in the operant trials as compared to the trials in which only the tone is presented, then it is concluded that outcome binding has occurred. Note, however, that in that methodology, the action binding effect, which is usually attributed to the delay between action and outcome, could also be attributed, at least in principle, to the mere presence of external feedback in the operant condition and not in the control ones.

In order to avoid this problem we will use only two different types of trials, and both of them will receive external feedback. They will differ only in the length of the action-outcome delay, so that we can ensure that the result is due to the differential delay and not to the presence of external feedback. In one trial type the tone will be delayed, in the other one, the tone will occur immediately after the action. Moreover, using this type of control condition, the only difference between action binding and outcome binding should reside in whether we ask participants to estimate the time at which the action occurred or the time at which the outcome occurred. Nevertheless, and because we are planning to test several other variables in these experiments and because asking both questions in each trial to the same participants could have a detrimental effect, we will focus only on the action binding effect in the present experiments. In this way, if a difference is observed between the estimated time of action in the delayed and the immediate condition, then we could conclude that the difference is due to the delay rather than to the mere presence of the tone. It is important to note that this kind of design has been previously used in other studies (e.g., Banks and Isham, 2009; Rigoni et al., 2010; Garaizar et al., 2016; Matute et al., 2017).

## Overview of the Experiments

The main goal of these experiments is to explore how certain methodological changes in Labclock Web could influence the temporal binding effects. Thus, the results of these experiments would help expand the potential use of this tool and therefore contribute to the advancement of new theoretical debates. In Experiment 1 we will reduce the delay between action and tone in the delayed condition to 100 ms [instead of 250 ms, as in Haggard et al. (2002a), or 500 ms, as in Garaizar et al. (2016)]. In Experiment 2 we will present the two types of trials, delayed and immediate, in separate blocks, instead of in randomized order. Finally, In Experiment 3 we will use a visual, instead of auditory, outcome. In addition, we tested one more transversal manipulation in the three experiments: we reduced the number of trials in each condition. Obtaining a temporal binding effect with a lower number of trials will also allow researchers to conduct experiments with limited resources and, most importantly, will reduce the number of participants who start the task but do not complete it due to boredom or fatigue.

## Ethics Statement

Participants for the three experiments were recruited through social networks. They did not receive any reward for their participation. The computer program informed participants that their participation was voluntary and anonymous. We did not ask participants for any data that could compromise their privacy, nor did we use cookies or software in order to obtain such data. The stimuli and materials were harmless and emotionally neutral, the goal of the study was transparent, and the task involved no deception. According to the U. S. Department of Health and Human Services (2009), as well as the American Psychological Association [APA] (2002), no written informed consent is required under these circumstances. Therefore we did not ask for consent, and so volunteers did not need to identify themselves. The Ethical Review Board of the Universidad a Distancia de Madrid examined and approved the procedure used in this research (CE-UDIMA/2017/CPC/0909007). The three experiments were conducted in accordance with the approved guidelines.

## Experiment 1

The duration of the action-outcome interval in the delayed condition has varied in temporal binding studies. For example, Buehner and Humphreys (2009) tested delays of 500, 900, and 1,300 ms whilst Cravo et al. (2013) used intervals of 250, 300, 350 ms and Wen et al. (2015) used 0, 100, 200, 300, 500, 700, and 1,000 ms delays between action and outcome. Nonetheless, the 250 ms action-outcome interval used by Haggard et al. (2002a) is the most standard delay used in the temporal binding literature, and is either used as a single delayed interval, or intermixed with other delays (e.g., Wolpe et al., 2013; Ruess et al., 2017). Moreover, an interval of 250 ms between action and outcome appears to cause a larger temporal binding effect than other intervals (Haggard et al., 2002a; Ruess et al., 2017).

Labclock Web (Garaizar et al., 2016) has so far been tested only with a 500 ms interval for the delayed condition, which is a much longer interval than the standard interval of 250 ms. The reason for this was simply that, at least in principle, the longer the intervals, the greater should be the accuracy of the task, and this is important if we would like this open-source software to become less sensitive to errors and to web-based noise and distractions. Moreover, Wen et al. (2015) reported that longer intervals produce stronger binding effects. Thus, we considered that experimental evidence showing that Labclock Web would also be able to detect temporal binding effects with shorter delays was a first and necessary step to confirm the reliability and generality of this tool. Therefore, the first purpose of Experiment 1 was to explore whether Labclock Web was an adequate tool for exploring temporal binding effects using delays that were much shorter than the 500 ms interval used by Garaizar et al. (2016) and even shorter than the 250 ms (more standardized) interval used by Haggard et al. (2002a). To this end we used a shorter interval of 100 ms in the delayed condition.

Also, we reduced the number of trial presented of each condition, from 40 to 10. That was conducted mainly because previous research in our laboratory had shown that indeed, binding effects were stronger during early training, with participants learning to better adjust their responses to the actual timing of their actions as they gain experience with the task (see Matute et al., 2017). In addition, this reduction in the total number of trials was desirable in order to reduce the duration of the task and, therefore, to reduce the number of participants who start the experiment but do not complete it. This is particularly important in online experiments in which participants are anonymous and voluntary Internet users who do not receive any reward (other than a brief explanation at the end of the experiment) in exchange for their participation.

### Materials and Methods

#### Participants

Thirty nine anonymous Internet users volunteered to participate in the experiment. The data of two participants were discarded because they emitted the action on <75% of trials in one of the two conditions. This criterion has been used in previous experiments (e.g., Garaizar et al., 2016; Matute et al., 2017).

#### Procedure

After reading the instructions, participants were given 10 trials with each of two experimental conditions. The two conditions differed in terms of the length of the interval between the action and the tone: immediate (1 ms) and delayed (100 ms). The order of presentation of the 20 trials was randomized for each participant. Each trial was announced by a 1,000 ms pre-trial tone compounded by two consecutive frequencies of 250 and 400 Hz. After a random interval between 0 and 2,000 ms the dot appeared and it started to rotate. Each revolution of the dot was completed in 2,560 ms. Participants were instructed not to give any response during the first revolution and to press the spacebar at any time of their choosing during the second revolution. Garaizar et al. (2016) had already tested the accuracy of stimulus presentation timing using Labclock Web, by monitoring the actual timing using an external tool. In the present experiments, we made use of their software so that, on all trials the action was followed by the tone, either immediately or after a 100 ms delay. In both conditions the duration of the tone was 200 ms and its frequency 1,000 Hz. When the second revolution ended the dot disappeared and the sentence “*Where was the dot when you pressed the spacebar?*” was presented at the top of the screen. Participants had to click in the clock-face to give their judgment, upon which a new trial began.

To summarize, although the main difference in this experiment with respect to that conducted by Garaizar et al. (2016) was the length of the interval in the delayed condition (100 ms instead of 500 ms) the design also differed in two minor aspects: (a) it included only 10 trials per condition instead of 40, and, (b) as discussed above, it did not include any post-experimental trials in which the action was not followed by the tone.

### Results and Discussion

The dependent variable was computed as the difference between the participants’ judgment of the time at which their action occurred and the actual time of action (T_judgment_ – T_action_). As expected, participants estimated that their action occurred later on the delayed trials (*M* = –12.41, SEM = 12.25) than on the immediate trials (*M* = –37.02, SEM = 6.53). A paired-samples t-test confirmed this impression *t*_(36)_ = 2.326, *p* = 0.026, *d_z_* = 0.38. These results suggest that Labclock Web is sensitive enough to detect temporal binding effects even when using very short intervals of 100 ms between action and tone and when reducing the number of trials to 10 per condition, which is convenient, particularly when performing the experiments with anonymous Internet users who will abandon them rapidly if they become bored or tired. However, besides this difference between groups, we found negative results in both groups showing that participants displaced their judgments away to the effect. We discuss this in General Discussion.

## Experiment 2

There have been many reports in the experimental psychology literature showing that previously acquired knowledge and expectations can affect learning and behavior at a future time. For instance, previous experience with a given type of trial, or set of trials, can affect responding on subsequent trials. That is, the order with which different trial types are presented can sometimes significantly affect the results. A well-known case in which this effect was reported is the Stroop task (Stroop, 1935). When a congruent trial is presented after another congruent trial in this task, the response is faster than when it is presented after an incongruent trial (Gratton et al., 1992). Moreover, in the causal learning literature, it has been reported that the order in which positive and negative instances of a cause-effect relationship are presented during the training session can have a profound impact. For instance, participants will come to judge that there is a stronger causal relationship when the positive instances are presented last than when they are presented first or in a randomized order (Collins and Shanks, 2002; Matute et al., 2002). In addition, it has been shown that there is also a cumulative effect of previous training, as can be seen, for example, in the decreasing attention that participants pay to the context when it provides none of the information needed to solve a task (León et al., 2010; Aristizabal et al., 2016). Classical experiments on proactive and retroactive interference are also excellent examples of the potential influence of trial order effects (Slamecka and Ceraso, 1960; Underwood, 1966).

In the temporal binding literature, trial order effects have been, to the best of our knowledge, scarcely studied. For example, Haering and Kiesel (2015; see also Haering and Kiesel, 2016) trained their participants with an action-effect delay of 0 ms in the immediate group, or 250 ms in the delayed group, during the first phase of their experiment. Then, during a second phase, this initial training was found to induce differences in the sense of agency about the effects that followed the action with different delays (0, 50, 100, 150, 200, and 250 ms). More specifically, participants perceived a greater sense of agency (i.e., they reported feeling more control) when the delays that were closer to those used in their previous training phase (0 or 250) and a weaker sense of agency (i.e., less control) when the intervals differed most from their initial experience. These authors found that the expectancy of the time of occurrence of the outcome, which was developed during Phase 1, affected the sense of agency in Phase 2. In addition, in a recent study, Matute et al. (2017), using Labclock Web, showed that participants became more accurate in their estimations of the time of their actions as they gained experience. As in the study of Haering and Kiesel, those results suggests that the judgments of the participants are affected not only by the specifics of each particular trial, but also by previous trials and the knowledge and expectations previously acquired in the task.

Haering and Kiesel (2015) divided their experiment into two different phases. In the first phase they presented only the delayed or the immediate trials, depending on the group. The other type of trial was presented in the second phase. However, they did not use the clock methodology, so we are uncertain as to whether their findings would generalize to Libet’s clock experiments. In contrast, Matute et al. (2017) conducted their experiments with the clock methodology but they presented the two types of trials in an intermixed manner. Thus, the aim of Experiment 2 was to explore whether the results of Haering and Kiesel (2015) would generalize to Libet’s clock experiments, using Labclock Web. That is, we aimed to test whether experiencing the action that causes the effect immediately or after a given time interval, has an impact on subsequent trials where the outcome is delayed or immediate. We expected to find a stronger temporal binding effect in the delayed condition when participants had previously experienced the immediate condition. In addition, we also intended to test whether the learning effect reported by Matute et al. (2017) can be observed when the trials are presented in different blocks (rather than intermixed).

### Materials and Methods

#### Participants

Sixty seven anonymous volunteers recruited through the Internet took part in this experiment. They were randomly assigned to one of two groups: immediate – delayed group (ID), with 35 participants, and delayed – immediate (DI) group, with 32 participants. According to the data selection criterion applied in Experiment 1, data from 4 participants from group ID and 1 from group DI were excluded from the analyses.

#### Procedure

In order to maximize the possible effect of the order of trials, 20 trials of each type were presented (instead of the 10 trials per condition that we used in Experiment 1). When participants accessed the experiment they were randomly assigned to one of two groups. In group ID all of the 20 immediate trials were presented at the beginning of the task and, after that, the 20 delayed trials were presented. In Group DI the order was reversed. Participants were not informed about the difference between the two blocks of trials and there were no breaks between blocks. To maximize discriminability as well as the potential effect of the immediate condition on the delayed condition, the tone was presented 500 ms after the action in the delayed condition (instead of 100 ms as used in Experiment 1). The tone appeared 1 ms after the action in the immediate condition. Other aspects of the procedure were identical to those described for Experiment 1.

### Results and Discussion

**FIGURE 1:**
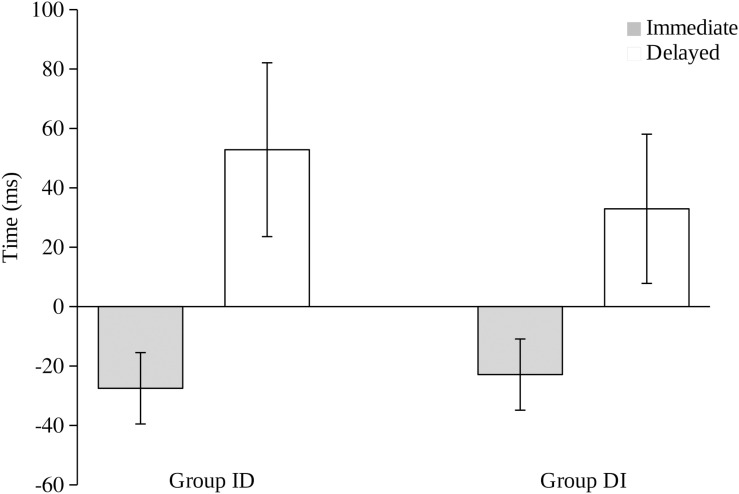
Difference (in ms) between the subjective judgment of the time of action and the actual time of action on the immediate (1 ms) and delayed (500 ms) trials in Experiment 2 for Group ID (immediate – delayed) and Group DI (delayed – immediate). A positive value means that participants estimated that their action occurred later than it did, and a negative value means that they estimated their action to have occurred earlier than it did. Error bars represent the standard error of the mean.

As in Experiment 1, we calculated the difference between the participants’ judgment of the time of their action and the actual time of action. The mean of these scores for both groups are shown in Figure 1, with the actual time of action represented as zero. Visual inspection of this figure suggests that the action was judged to have occurred later in the delayed condition than in the immediate condition in both groups. A 2 (group: ID, DI) × 2 (condition: immediate, delayed) ANOVA revealed a main effect of condition, *F*_(1, 60)_ = 14.591, *p* < 0.001, η_p_^2^ = 0.196, but not a main effect of group (ID vs. DI), *F*_(1, 60)_ = 0.103, *p* < 0.750, η_p_^2^ = 0.002, or an interaction between these variables, *F*_(1, 60)_ = 0.550, *p* < 0.461, η_p_^2^ = 0.09. *Post-hoc* analyses showed differences between the immediate and delayed conditions in both groups, *t*_(30)_ = 2.869, *p* = 0.007, *d_z_* = 0.51 for Group ID and *t*_(30)_ = 2.558, *p* = 0.016, *d_z_* = 0.46 for Group DI. Thus, we can conclude that, using the clock methodology and presenting the trials ordered in blocks by condition, the temporal binding effect was also observed, as in the case in which the different trials are presented in an intermixed manner (e.g., Garaizar et al., 2016; Matute et al., 2017). Moreover, this occurs regardless of whether the participants receive the immediate or the delayed trials first or second. Thus, we replicated the binding effect using a block presentation of trials but did not replicate the trial order effect according to which the expectation acquired during a first phase should affect the judgments of the second phase.

**FIGURE 2:**
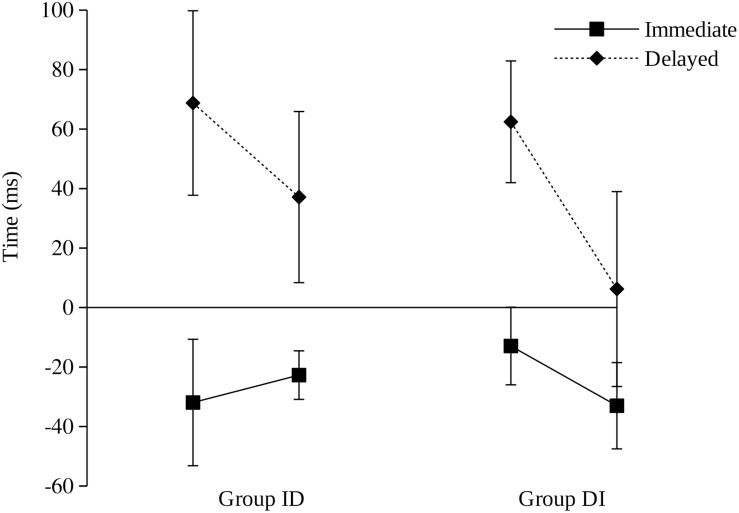
Mean of the time estimated action minus the actual time of action for immediate (1 ms) and delayed (500 ms) trials in Experiment 2 for Group ID (immediate – delayed) and Group DI (delayed – immediate). A positive value means that participants estimated that their action occurred later than it did, and a negative value means that they estimated their action to have occurred earlier than it did. Error bars represent the standard error of the mean. Each data point represents the first 10 (B1) and the last 10 trials (B2) of each condition. Error bars represent the standard error of the mean.

However, it could be argued that the effect of the order of trials should not be observed when the data of the individual trials are averaged. According to Matute et al. (2017), the judgments should be changing as participants gain experience, although it should also be noted that their experiments included a large number of intermixed trials, so we are uncertain about how learning and experience should affect judgments in our current experiment, in which we are using independent phases composed of different and fewer trials. To test this idea, as well as to test whether participants actually modified their judgments with experience, the 20 trials of each condition were split into two blocks of 10 trials each: B1 and B2. Figure 2 shows the participants’ judgments in the immediate and the delayed condition in each of these blocks. B1 represents the mean of the first 10 trials of each condition and B2 the mean of the 10 last trials. A visual inspection of this figure shows that the participants became more accurate as training proceeded in both groups in the delayed condition. This effect, however, does not appear to occur in the immediate condition. A 2 (group: ID, DI) × 2 (condition: immediate, delayed) × 2 (block: B1, B2) ANOVA revealed a main effect of condition *F*_(1, 60)_ = 14.931, *p* < 0.001, η_p_^2^ = 0.199 and a main effect of block *F*_(1, 60)_ = 6.557, *p* = 0.013, η_p_^2^ = 0.199, but no effect of group *F*_(1, 60)_ = 0.088, *p* = 0.767, η_p_^2^ = 0.001 or a triple interaction between these variables *F*_(1, 60)_ = 0.023, *p* = 0.880, η_p_^2^ < 0.001. Paired comparisons between the two blocks in each condition revealed significant differences between B1 and B2 in the delayed condition, *t*_(30)_ = 2.517, *p* = 0.017, *d_z_* = 0.45 for Group ID and *t*_(30)_ = 2.577, *p* = 0.015, *d_z_* = 0.46 for Group DI, but not in the immediate condition, *t*_(30)_ = 0.447, *p* = 0.658, *d_z_* = 0.08 for Group ID and *t*_(30)_ = 1.466, *p* = 0.153, *d_z_* = 0.26 for Group DI. These analyses confirm that participants learned to modify their judgments with experience, which replicates the results found by Matute et al. (2017). However, it seems that the order of presentation in the conditions did not impact this learning effect.

To sum up, we can conclude that Experiment 2 replicated the temporal binding effect using Labclock Web when presenting the different trial types separated in blocks by condition, rather than in an intermixed manner. Moreover, we replicated, under very different conditions, the learning effect described by Matute et al. (2017), showing also that the effect appears to be unaffected by the order of presentation. However, these results do not replicate the order effect shown by Haering and Kiesel (2015). In the General Discussion, we will put forward some factors that could account for this discrepancy.

## Experiment 3

The aim of the study by Garaizar et al. (2016) was to present Labclock Web as a tool that could offer researchers the possibility of conducting experiments based on Libet’s clock paradigm. Previous research, on many different topics, had shown that experiments conducted through the Internet could produce the same or very similar results as those conducted in the laboratory (McGraw et al., 2000; Gosling et al., 2004; Kraut et al., 2004; Vadillo et al., 2006; Germine et al., 2012). In fact, using Labclock Web, Garaizar et al. (2016) showed that temporal binding was observed both in laboratory experiments and in participants recruited through the Internet. However, an inherent problem of Internet-based experiments is that researchers have less control over factors that could affect the results, such as the configuration of the individual computers where the experiment is being conducted, and the environmental conditions, among several others.

The original clock procedure requires that participants have four external devices on their computers: screen, mouse, keyboard, and earphones (or speakers). In laboratory research, the experimenters can ensure that the computer has these four devices set up in optimal conditions. However, such control is lacking in online experiments. In temporal binding experiments, the most problematic of these four devices are the earphones or speakers. The reason is that the experimenters are not able to assess whether participants are conducting the task with earphones or with speakers, whether they have set them at the correct volume, or whether other external factors could be affecting correct perception of the auditory stimuli (e.g., participants might be carrying out the task in a loud place with distracting sounds).

With the aim of avoiding this potential problem caused by auditory stimuli in experiments conducted through the Internet, in Experiment 3 the outcome following the action was visual instead of auditory. More specifically, the experiment was programmed so that the color of the rotating-dot would change from black to red after the action. Visual feedback has been successfully used in some previous temporal binding experiments (e.g., Banks and Isham, 2009; Ruess et al., 2018a). The purpose of this experiment was to test whether Labclock Web would still allow to generate and detect the temporal binding effect if the auditory feedback were replaced by a visual feedback. Using visual feedback should in principle facilitate the reliability of Internet-based experiments on binding effects by providing more standardized conditions than auditory ones (which, as mentioned above are strongly affected by many different circumstances). However, it is important to test whether presenting cross-modal stimuli would affect the results because auditory and visual stimuli have sometimes produced different results (see Wearden et al., 2010; Hass et al., 2012; Allman et al., 2014).

### Materials and Methods

#### Participants

Thirty-seven anonymous Internet users volunteered to participate in this experiment, which was conducted online. Following the criteria described in Experiment 1 the data from nine participants were excluded from the analysis.

#### Procedure

The procedure was identical to that described in Experiment 1 except for two modifications. There was no auditory outcome following the action. Instead, the action was followed by a change in the color of the rotating-dot, which changed from black to red when the action was performed. The instructions of the experiment were changed to reflect this modification. Moreover, with the aim of replicating the results of Garaizar et al. (2016), with the only difference being the sensory modality of the outcome, the interval between action and feedback (i.e., color change) was 500 ms, as in Experiment 2. This change should facilitate the observation of binding in case the use of visual feedback produced some detriment. The number of trials was, as in Experiment 1, 10 per condition.

### Results and Discussion

As in Experiments 1 and 2, we calculated the difference between the actual time of action and the participants’ estimation of this time. As expected, the participants judged the time of their action as having occurred later when the outcome was delayed (*M* = 18.74, SEM = 22.41) compared to when it was presented immediately after the action (*M* = –21.24, SEM = 8.29). A *t*-test confirmed this impression *t*_(27)_ = 2.300, *p* = 0.029, *d_z_* = 0.43. This result confirms that a visual outcome, used instead of the standard auditory tone, could also generate a temporal binding effect in this paradigm. This should allow researchers to conduct experiments using visual outcomes, which allows for a more convenient and reliable presentation of the experiments through the Internet. As in Experiment 1, the number of trials in each condition was reduced to ten, in order to reduce the duration of the task. The results suggest that Labclock Web allows for the running of experiments with this low number of trials. This makes it easier to recruit voluntary participants and presumably implies a reduction of the number of people who start the experiment and do not finish it. This, again, is particularly important in experiments conducted through the Internet.

## General Discussion

Labclock Web (Garaizar et al., 2016) is a tool that allows for conducting experiments based on Libet’s clock through the Internet. One of the effects most frequently studied using this procedure is the temporal binding effect. The three experiments presented here tested several methodological modifications in this task and their impact on the temporal binding effect. The results confirm that Labclock Web is a useful tool for carrying out temporal binding experiments, not only when using the standard features and parameters that have already been published in the literature but also when (a) using short action-outcome delays (i.e., 100 ms), (b) presenting the different types of trials in separate consecutive blocks, (c) using visual instead of auditory feedback, and (d) reducing the number of trials per condition.

Experiment 1 shows that Labclock Web is able to detect the temporal binding effect when the interval between action and tone in the delayed condition is 100 ms, instead of 500 ms, as in Garaizar et al. (2016) or 250 ms as in Haggard et al. (2002a). Although the experiments described in the literature used a great variety of delays between action and tone, Labclock Web had only been tested with 500 ms, which might have favored the observation of the effect. It is important for researchers to ensure that the tool used to program and conduct the experiments is sensitive and homogeneous in the presentation of stimuli and in response recording. This is particularly important in experiments that use time as the relevant dependent variable. It is important to note that the aim of Experiment 1 was not to obtain temporal binding using shorter than 500 ms delays, what has been yet observed in previous studies (see e.g., Banks and Isham, 2009). Instead of that, the objective in this experiment was to test if Labclock Web IS an accurate tool to handle shorter than 500 ms delays. We found an unexpected negative effect in both, immediate and delayed (100 ms) condition, showing that participants judgments are displaced not toward but away from the outcome. This kind of negative effect is sometimes observed in the literature. For example, in Experiment 1 of Banks and Isham (2009), which was conducted using the Libet’s clock methodology, they delayed the feedback 5, 20, 40, or 60 ms from the action. They found that participants’ judgments were displaced −122, −104, −95, and −77 ms, respectively. Importantly, they asked participants to report the moment in which they decided to act, instead of the moment of the action. This could have contributed to those negative binding results, although the fact that they used very short delay intervals, as was also the case in our experiment, might also have been important. Other studies that used Libet’s clock methodology are also hardly comparable with Experiment 1, either because they used much longer intervals between action and effect (e.g., Wohlschläger et al., 2003; Engbert and Wohlschläger, 2007; Matute et al., 2017) or because they computed the dependent variable by subtracting the baseline condition (e.g., presenting no tone) from the regular trials (e.g., Haggard et al., 2002b; Moore et al., 2009). Therefore, we cannot be certain as to why this effect took place. Additional experiments would be needed to explore this effect by comparing, for example, conditions with delays of 100 vs. 500 ms. In any case, it should be noted that we are measuring temporal binding as the difference between conditions, and results showed that this difference was reliable, proving that this tool is sensitive enough to conduct experiments with short delays.

In Experiment 2 the two types of trials (delayed and immediate) were presented in two different blocks. All delayed trials were presented during the first phase, and all immediate trials were presented during the second phase in the DI group. This order was reversed in Group ID. The results suggest that the order of presentation of one or the other type of trial did not affect the temporal binding effect, which was replicated in both cases. Moreover, we also found a learning effect: participants adapted and modified their judgments as they gained experience with each phase. This learning effect had been previously described by Matute et al. (2017) for the entire experimental session, in which they had presented the different types of trials in an intermixed manner. We replicated this learning effect within each phase in the delayed condition, though not in the immediate condition. We also found that the order in which the trials are presented did not seem to impact this learning effect in the delayed condition, as it occurred both in the ID and the DI groups. However, Haering and Kiesel (2015) have shown that prior experience with a certain delay could affect the judgments on subsequent trials with different delays. We did not replicate their effect. Some reasons that could account for the different result we observed with respect to Haering and Kiesel are: (a) the difference in the task: they did not use the clock paradigm, and (b) the different judgments: they used a judgment of control, that is, they asked their participants to indicate to what degree they felt they were responsible for the occurrence of the outcome, whereas we used a judgment of when the event occurred. Thus, more experiments would be needed to elucidate under which conditions previous training affects subsequent judgments. It also seems reasonable to assume that our reduction in the number of trials might have weakened the development of the expectation that could otherwise have formed during the first phase. If this were the case and a strong expectation could not be formed, then there would be no reason why the order of trials should influence the second phase.

Although recent technological developments have facilitated the proliferation of Internet-based research, the conditions under which participants carry out the experiments are less controllable than in the laboratory. This lack of experimental control could affect participant’s responses through the presence/absence of visual or auditory distractors during the experiment or in critical technical aspects. For example, in experiments conducted in laboratory researchers could make sure that the screens’ refresh rate are identical in all computers. However, in experiments conducted through the Internet, each participant’s computer could have a different refresh rate. This could make the presentation of the stimuli less precise in screens with a lower refresh rate. For example, in screens with the lowest refresh rate (30 Hz) the image is refreshed each 33.33 ms. Thus, the presentation of a stimulus could be retarded, at most for this amount of time. This gap is minimized in high refresh rated screens (240 Hz) where images are refreshed each 4.16 ms. This difference is particularly relevant in between-participants’ designs, in which participants’ responses in different conditions could be differently affected by such factors, although, presumably, they should be randomly distributed across conditions. Thus, we are aware that it is not possible to be certain that all participants are conducting the experiment in the best conditions. For this reason, it is important to ensure that the factors that could affect the results of online experiments are minimized.

Experiment 3 aimed to reduce some of these factors in temporal binding experiments (i.e., the need to use earphones or speakers, the need for a proper setting of the volume, and the potential influence of environmental noise on the perception of auditory stimuli). Thus, we changed the modality of the outcome presentation to visual instead of auditory. In this experiment, the action caused immediately (in the immediate condition) or after 500 ms (in the delayed condition) a color change of the rotating dot from black to red. The results show that this configuration of the task is also well suited to promoting a temporal binding effect. However, it is important to note that in both delayed and immediate conditions, we cannot be certain of when participants perceived the presentation of the outcome. It could be argued that participants detected it exactly when the rotating dot changed its color or, alternatively, because the dot kept rotating in red until the termination of that cycle, they might have detected the change some time later. As mentioned, differences in the refresh rate of participants screens could also affect the accuracy of the color-change event. That implies that participants of Experiment 3 could experience the feedback (i.e., color change), not exactly at the moment in which it occurred, but sometime between the actual color change and some milliseconds after it. Nevertheless, this should have affected the two conditions equally, making participants experience the feedback in the delayed condition as occurring later than in the immediate condition. Indeed, the results show that, even with this limitation, Labclock Web was still capable of detecting a binding effect using this color change as the feedback signal. Perhaps more experiments are needed in which the outcomes are programmed as a temporal color change, from black to red and, after some tenths of seconds, again to black. Although we would expect the same results than in Experiment 3, this manipulation would be more similar to the traditional auditory presentation. Also further experiments are still needed to further test the accuracy of Labclock Web and its usability in additional conditions. For example, in our experiments we reported that participants judge their action as occurring later in the delayed condition (i.e., action binding). It could be interesting to test if Labclock Web is also suitable to measure whether participants judge the effect earlier (i.e., effect binding) as well (recall that our experiments were not designed to test for effect binding). Two additional manipulations that should be tested are the effect of the contingency between events (e.g., Buehner and Humphreys, 2009) and the effect of participant’s will (e.g., Haggard et al., 2002b). Finally, some studies that used Libet’s clock paradigm stop the rotating dot at an unpredictable time after the occurrence of the effect (e.g., Haggard et al., 2002b; Ruess et al., 2018b) instead of terminating rotation always in the same location as we do. Although, as far we know, there is no evidence suggesting that this could affect participants’ judgment, this could also be a new and interesting feature to incorporate in this tool.

As mentioned in the Introduction, it is important to note that we did not include baseline conditions in any of these experiments. Baseline conditions are composed by trials in which participants experience only one event (i.e., the action or the outcome) and they are asked to estimate the moment in which it occurs (e.g., Haggard et al., 2002b; Moore et al., 2009). Although these baseline conditions could be informative because they reveal the accuracy of participants’ estimation in the absence of delay, we decided not to include them and to substitute them for the immediate conditions. These control (immediate) conditions, received the same outcomesas the operant conditions and differed from them only in that the outcomes were presented immediately after the actions rather than after a delay. Thus, we believe these immediate conditions should offer better control of the accuracy of the participants’ estimation of the time of action in the absence of delay than the standard baseline conditions. Indeed, the only difference between our two conditions is whether there is a delay between action and outcome, and not whether the outcome is present or absent.

In sum, the contribution of the present experiments can be summarized in three main points. Firstly, they add support to a growing line of literature showing that the temporal binding effect is a robust phenomenon that can be replicated under a variety of conditions (Haggard et al., 2002a; Mitchell et al., 2005; Stetson et al., 2006; Zhao et al., 2013; Ruess et al., 2018b, see also, Moore and Obhi, 2012). Secondly, they provide empirical support for a theoretical approach of the binding effect. For example, we replicate here the learning effect found by Matute et al. (2017), and this could have a deep impact on how the binding effect is explained. Finally, the three experiments presented here, together with those presented by Garaizar et al. (2016) and Matute et al. (2017), confirm the usefulness of Labclock Web, and confirm that this tool can be reliably used in a variety of situations in which the Libet’s clock procedure is used. In this age of replication crisis in psychology and need for transparency and reproducibility, we believe that the present results are of considerable interest in supporting the reliability of open source software such as Labclock Web, which can be easily programmed (and audited) by any researcher worldwide, whilst also being easy to share and reproduce. Also given that this tool is freely downloadable as an Open Source tool, it does provide the homogeneous environment that is necessary for the reliability and reproducibility of research which is needed nowadays. Finally, the present experiments have provided further evidence of the range of conditions under which this tool can work in an adequate manner.

## Data Availability Statement

The dataset for this study can be found in the Open Science Framework at the following URL: https://osf.io/avfbk/?view_only=a4e7146f34494e3d86740d20103e80c8.

## Ethics Statement

The studies involving human participants were reviewed and approved by the Ethical Review Board of the Universidad a Distancia de Madrid: CE-UDIMA/2017/CPC/0909007. The study was anonymous, voluntary, and harmless. Written informed consent for participation was not required for this study in accordance with the national legislation and the institutional requirements.

## Author Contributions

ÍL developed the software and conducted the experiments. ÍL and CC analyzed the data. CC drafted the manuscript. ÍL and HM provided critical revisions. All authors developed the study concept and approved the final version of the manuscript for submission.

## Conflict of Interest

The authors declare that the research was conducted in the absence of any commercial or financial relationships that could be construed as a potential conflict of interest.
